# Study on the Occurrence Characteristics of the Remaining Oil in Sandstone Reservoirs with Different Permeability after Polymer Flooding

**DOI:** 10.3390/polym16131902

**Published:** 2024-07-02

**Authors:** Xianda Sun, Limin Suo, Yuanjing Huang, Hongyu Wang, Han Yu, Chengwu Xu, Jian Xu, Xudong Qin, Wenying Sun, Yangdong Cao, Tao Liu

**Affiliations:** 1 National Key Laboratory of Continental Shale Oil, Northeast Petroleum University, Daqing 163318, China; sunxianda@nepu.edu.cn (X.S.); 15192918765@163.com (Y.H.); 18536066626@163.com (H.W.); yyhh98917@163.com (H.Y.); 15512183313@163.com (J.X.); 228003010609@stu.nepu.edu.cn (X.Q.); swenying2022@163.com (W.S.); cyangdong0221@163.com (Y.C.); nn15938610741@163.com (T.L.); 2 College of Information and Electrical Engineering, Heilongjiang Bayi Agricultural University, Daqing 163319, China; suolimin@byau.edu.cn

**Keywords:** polymer flooding, residual oil distribution, permeability, micro-CT, pore structure

## Abstract

After polymer flooding, the heterogeneity between different layers intensifies, forming intricate seepage channels and fluid diversions, which results in decreased circulation efficiency and lower recovery rates, leaving a significant amount of residual oil trapped within the reservoir. Understanding the characteristics of residual oil occurrence is crucial for enhancing oil recovery post-polymer flooding. This study focused on sandstone reservoirs with varying permeability in the Saertu block of the Daqing oilfield. Using cryosectioning and laser scanning confocal microscopy, the occurrence characteristics of the residual oil in these sandstone reservoirs post-polymer flooding were investigated. Additionally, micro-CT and scanning electron microscopy were employed to analyze the impact of the pore structure on the distribution characteristics of the residual oil. The results indicate that laser scanning confocal images reveal that post-polymer flooding, the residual oil in high- and low-permeability sandstone reservoirs predominantly exists in a bound state (average > 47%), mostly as particle-adsorbed oil. In contrast, the residual oil in medium-permeability reservoirs is primarily in a free state (average > 49%), mostly as intergranular-adsorbed oil. In high-permeability sandstone reservoirs, heavy oil components are mainly in a particle-adsorbed form; in medium-permeability sandstone reservoirs, residual oil predominantly consists of heavy components, with most light components occurring in a clustered form; in low-permeability sandstone reservoirs, clustered residual oil exists in a balanced coexistence of light and heavy components, while the heavy components primarily exist in a particle-adsorbed form. Post-polymer flooding, the large pore–throat structure in high-permeability sandstone reservoirs results in effective displacement and less free residual oil; medium-permeability sandstone reservoirs, with medium–large pores and throats, have preferential channels and fine particles blocking the throats, leading to some unswept pores and more free residual oil; low-permeability sandstone reservoirs, with small pores and throats, exhibit weak displacement forces and poor mobility, resulting in more bound residual oil. The distribution and content of clay particles and clay minerals, along with the complex microscopic pore structure, are the main factors causing the differences in the residual oil occurrence states in sandstones with varying permeability.

## 1. Introduction

The Daqing oilfield, as one of the largest onshore oilfields in China, has undergone extensive development and production. Currently, it faces the challenge of declining recovery rates year by year. With the oilfield entering a high water-cut stage, traditional extraction methods can no longer meet the demand, and the effective development of the residual oil resources has become an urgent issue. In this context, polymer-flooding technology has garnered significant attention [1,2]. Currently, polymer flooding is the most commonly used chemical-enhanced oil recovery method worldwide. Polymer flooding improves the water injection viscosity, enhances the sweep efficiency, and reduces the channeling in high-permeability pathways, thereby significantly improving the oil recovery efficiency [3,4,5]. This technique has become a key method for enhancing recovery rates and exploiting the residual oil potential, especially in mature oilfields with high water cut, where it holds substantial application value [1,6]. Zhao et al. [7] improved the reservoir recovery by 56.9% through the in situ emulsification and migration control mechanism by lowering the concentration of the surfactant–polymer system. Wei et al. [8] suggested that surfactant–polymer flooding can increase reservoir recovery by approximately 30% compared to water flooding. Yu et al. [9] emphasized that surfactant polymers can reduce interfacial tension, and their emulsification performance and water-wettability trend are crucial for enhancing microscopic displacement efficiency. Zhang et al. [10], using the Dagang oilfield as an example, found that polymer–surfactant flooding can increase the oil recovery efficiency by 12–29% post-polymer flooding. However, the physicochemical properties of polymers significantly influence the displacement efficiency. Recent studies by Mursalhe et al. [3] indicated that besides viscosity, the viscoelasticity of polymers can effectively mobilize residual oil and enhance the microscopic displacement efficiency, thereby increasing the oil recovery and further improving the macroscopic sweep efficiency. Olabode et al. [11] found that high salt concentrations (10% by weight) in polymers or reservoir fluids reduce oil recovery during polymer flooding. Zene et al. [12] highlighted that the viscosity, polymer capture, residual detritus in the reservoir, polymer rheology, mechanical degradation, and permeability reduction are major factors influencing polymer injection rates.

The aforementioned studies indicate that enhancing polymer properties can increase reservoir recovery rates. However, a substantial amount of oil remains in the reservoir post-polymer flooding [13,14,15,16]. Understanding the distribution characteristics of residual oil in rocks can help identify high-permeability channels and low-permeability areas within the reservoir, optimize injection strategies, reduce ineffective cycles, and increase the sweep area and volume of the displacement agent within the reservoir. Previous studies have utilized digital core technology, nuclear magnetic resonance (NMR) technology, and fluorescence analysis to characterize the residual oil distribution and occurrence states [17,18,19,20,21,22]. Fang et al. [23] used a technique to categorize post-waterflood residual oil into five types: cluster, oil film, oil droplets, columnar, and blind-end residual oil, all of which can be activated by polymer flooding. Zhang et al. [24] using cryo-fluorescence thin-section technology, classified microscopic residual oil into three types: free-state residual oil, semi-free-state residual oil, and bound-state residual oil. Li et al. [25] characterized residual oil post-polymer flooding using laser scanning confocal fluorescence microscopy, a technique that provides clearer images to distinctly separate oil–water interfaces and visually display mineral morphology and microscopic residual oil distribution. However, studies on the occurrence state of residual oil in sandstone with different permeabilities are limited.

Numerous studies have shown that the pore structure of rocks is a critical factor affecting the residual oil characteristics [26,27]. Yang and Ge et al. [28] used NMR to study the distribution of oil and water in cores and demonstrated a close relationship between the residual oil distribution and pore structure characteristics. Sun et al. [29] suggested that the distribution of residual oil is influenced by multiple factors, including the microstructure relationship. Gong et al. [30] found that the difference in pore structure controlled by sedimentary structures is the main reason for the reservoir microscopic heterogeneity in sandstone reservoirs. The residual oil layers are distributed in sheet-like forms, enriched in the upper part of the composite rhythm layers, influenced by the uneven creeping of the displacement fluid along large pore–throat channels. Micro-CT can non-destructively provide high-resolution three-dimensional images of the interior of rocks, visually characterizing the pore size and connectivity, aiding in predicting reservoir fluid flow behavior [31,32,33,34,35]. Recently, Kumar and Georgiadis et al. [36,37,38] proposed various applications of digital cores in studying displacement effects and quantitatively characterizing the residual oil distribution. Elkatatny and Rücker et al. [36,39] captured three-dimensional images of reservoir rock pore structures and internal residual fluids with micrometer resolution, maintaining cluster movement through a series of breakage and merging processes in capillary-dominated flow states. Li et al. [40], using advanced image processing technology, achieved precise separation of water, oil, and particles, established a pore network model, calculated parameters such as the throat number and throat size distribution, and characterized the heterogeneity of microscopic pore structures. When the heterogeneity is strong, the aqueous phase preferentially flows through dominant paths and residual oil clusters form in small pores.

Polymer-flooding technology has been extensively applied in the Daqing oilfield during its construction period. In the evaluation area, six polymer-engineering methods and ten technologies applicable to polymer flooding have been established, forming a comprehensive research capability and field support technology for polymer flooding. Additionally, from 1996 to 1998, the Daqing oilfield implemented polymer flooding in 10 blocks, encompassing 1753 wells, with an annual oil production of 8.1691 million tons, accounting for one-seventh of the Daqing oilfield’s annual output [41]. This study selected samples from the Saertu block of the Daqing oilfield after polymer flooding. In 2009, the five-spot method was used for polymer-flooding development in this area, with a basic well spacing of 175 m. The crude oil currently has a wax content of 22.02%, a gum content of 14.80%, a pour point of 32.88 °C, and an oil density of 0.8690 g/cm^3^. The rock compressibility coefficient is 9.4 × 10^−5^/MPa, and the reservoir temperature is 49 °C. As of December 2020, the average daily fluid production per well is 27 tons, with an average daily oil production of 1.3 tons and a comprehensive water cut of 95.2%. The block utilizes polyacrylamide produced by the Daqing oilfield, with a designed injection molecular weight of 1200 to 1600 × 10^4^ Da. The injection concentration is fully matched to the development status of each well, with an injection rate of 0.15 to 0.2 PV/a, an average injection concentration of 1200 mg/L to 1300 mg/L, and a wellhead injection viscosity of 50 mPa·s. Compared to water flooding, the recovery rate has improved by 15–20%. The main issue currently affecting polymer flooding in this area is the viscosity loss rate. The target formation of the block is primarily composed of fine sandstone and siltstone deposits; the formation water is NaHCO_3_-type, weakly alkaline, with a salinity of 5559.38 mg/L.

Post-polymer flooding, a substantial amount of residual oil remains in the sandstone reservoirs of the Saertu block in the Daqing oilfield. To clarify the microscopic residual oil distribution characteristics within the sandstone reservoirs of the Saertu block and provide suggestions and directions for adjusting the polymer-flooding schemes for different permeability reservoirs and different types of residual oil, this study employed the following methods: (1) utilizing cryosectioning technology and laser scanning confocal microscopy to quantitatively analyze the microscopic distribution characteristics of the residual oil in sandstone reservoirs with different permeabilities post-polymer flooding; and (2) conducting scanning electron microscopy and micro-CT experiments to analyze the pore distribution characteristics of sandstone with different permeabilities, studying the impact of the pore structure on the occurrence state of residual oil.

## 2. Materials and Methods

### 2.1. Characteristics of Sandstone Samples

To ascertain the occurrence states and distribution patterns of residual oil in reservoirs with varying permeabilities, six sandstone core samples were selected for thin-section preparation. Core samples 1 and 2 were high-permeability samples, core samples 3 and 4 were medium-permeability samples, and core samples 5 and 6 were low-permeability samples. The specific physical properties of these sandstone samples are detailed in Table 1. Utilizing Leica K8 laser scanning confocal microscope produced by German Leica company, the microscopic occurrence states and distribution of residual oil were visually and quantitatively characterized, with the experimental testing temperature set at 24 °C.

### 2.2. Thin-Section Preparation

**Figure 1:**
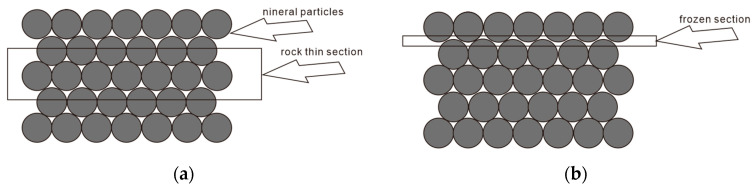
Core preparation methods. (**a**) Conventional core preparation. (**b**) Cryogenic core preparation.

To minimize the dissipation of oil and gas from the formation cores, the freshly extracted cores were cryogenically preserved using liquid nitrogen freezing technology. Once fully frozen, the cores were retrieved and sliced into sections with a diameter of 10 cm and a thickness of 2 cm. To ensure observational accuracy under the microscope, the natural fracture surfaces of the rock samples were selected during the sectioning process. Use non fluorescent resin adhesive produced in Daqing Oilfield, China for bonding, and then polish to a thickness of 0.05mm.Compared to conventional thin sections with a diameter of 2.5 cm, this study expanded the diameter to 10 cm and reduced the thickness from 1 mm to 0.05 mm. This approach enabled extensive observation of the pore structure and residual oil within the rock samples, enhancing the precision twenty-fold, facilitating better identification of oil, water, and rock particles, and avoiding the interference of multi-layer particle overlap and upper and lower layer pores, as shown in Figure 1.

### 2.3. Laser Scanning Confocal Experiment

The laser scanning confocal experiment utilized a Leica SP8 laser scanning confocal microscope from Germany. Compared to conventional fluorescence microscopes, the laser scanning confocal microscope employs a point light source and pinhole aperture to avoid light-scattering interference. The ultraviolet laser light source offers higher resolution (0.18 μm), increased brightness, and stable intensity, resulting in clearer images that reveal differences in the crude oil distribution. The specific colors of crude oil under ultraviolet light are shown in Table 2. Bituminous oil appears yellow, yellow–white, or pale yellow; aromatics are blue, blue–white, or light blue; and formation water, which dissolves a small amount of aromatics, appears light blue in fluorescence images. Thus, under ultraviolet excitation, the luminescent properties of crude oil at different locations can be utilized to quickly and accurately calculate the oil saturation, ultimately determining the absolute proportions of rock, oil, and water within the core thin sections.

**Figure 2:**
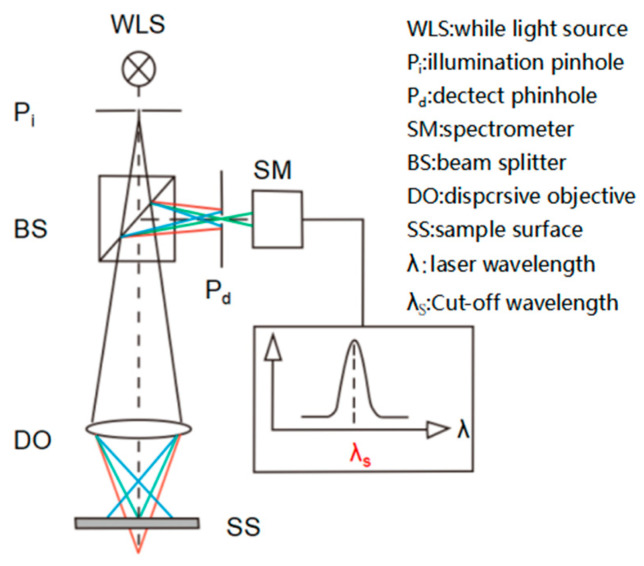
Schematic diagram of the principle of a confocal laser scanning microscope.

Imaging Principle of Laser Scanning Confocal Microscopy: The laser beam passes through a pinhole aperture and is focused on each minute point of the sample. The emitted light signals then pass through a pinhole placed in the emission light detection path, reaching the detector. The focal plane of the objective lens is conjugated to the positions of the detection pinhole and the incident light source pinhole, thus light from above and below the focal plane is blocked at the pinhole, while light from the focal plane can pass through and be detected. The incident light spot scans the sample point by point or line by line in the focal plane (*xy* axis) perpendicular to the microscope’s optical axis, with the scanning information being analyzed and processed into a two-dimensional image by a computer. The two-dimensional images of different focal planes obtained from scanning serve as “sliced” images within the focus depth range. By processing these multiple xy-plane images scanned at different *z*-axis positions along the microscope’s optical axis with a computer, one can obtain a comprehensive view, as depicted in Figure 2.

### 2.4. Three-Dimensional Image Reconstruction Technology

When employing laser confocal microscopy for imaging samples, the process centers around a minute light source point. This system achieves high-precision imaging by accurately scanning the sample’s surface point by point or line by line in planes parallel to the microscope’s optical axis (xy plane). During scanning, the image data captured at each point are sequentially recorded and processed by a computer, ultimately merging into a complete two-dimensional image. This step effectively captures a cross-sectional image of an exceedingly thin region of the sample. By repeating this process at different depths (along the *z*-axis), multiple two-dimensional sectional images are obtained. These two-dimensional sectional images are then overlaid and combined using computer software, reconstructing the sample’s three-dimensional structure. This three-dimensional imaging capability of the laser confocal microscope is exceptionally suited for in-depth exploration of the three-dimensional distribution of oil, water, and rock in oilfields.

### 2.5. Fluorescence Analysis of Residual Oil

**Figure 3:**
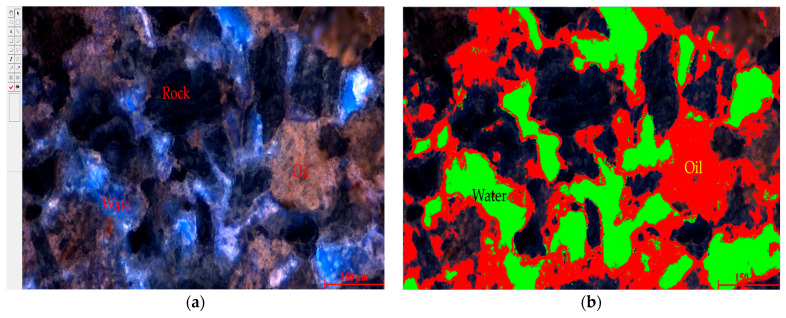
Fluorescence image segmentation. (**a**) Original fluorescence image. (**b**) Image segmentation result.

**Figure 4:**
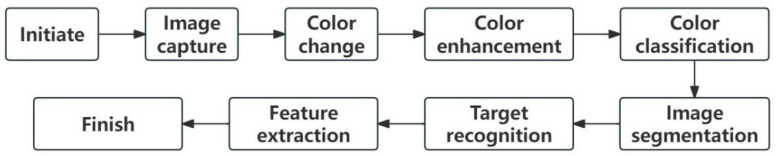
Fluorescence analysis workflow for residual oil.

For the analysis of the residual oil occurrence states, a proprietary fluorescence analysis software is utilized. In fluorescence images, crude oil appears yellow–brown, water is blue, and rock is gray–black, as shown in Figure 3a. Threshold segmentation based on image characteristics isolates the desired targets from the whole, where red indicates crude oil and green indicates water, as shown in Figure 3b. After segmentation, the software automatically calculates parameters such as the oil-bearing area, water-bearing area, and the proportion of different types of residual oil. The specific process is illustrated in Figure 4.

### 2.6. Scanning Electron Microscopy Experiment

The experiment utilized a ZEISS Gemini SEM 300 field emission scanning electron microscope from Germany. Prior to the experiment, relatively flat natural fracture surfaces were selected, cleaned of surface contaminants, and gold-coated in a vacuum environment. The high vacuum mode was used with a working distance of 3–15 mm, voltage range of 1–15 kV, and current range of 0.05–0.3 nA, achieving a resolution of 5–50 nm. This setup ensured clear images with a good signal-to-noise ratio, appropriate brightness, and appropriate contrast, meeting the required resolution standards. To ensure that the microstructural characteristics representative of the reservoir were observable, each sample was sectioned into 1 cm^3^ cubes, and 2–3 regions were selected for gradual observation from a low-magnification overview to a high-magnification microstructure.

### 2.7. Micro-CT Digital Core Technology

Using the Sanying NanoVoxel-3502E CT Scanner in Tianjin, China.The sandstone samples were meticulously placed at the center of the CT machine’s rotating stage, ensuring precise alignment with the scanning area. The scanning conditions were set at a voltage of 120 kV, a current of 120 µA, and an accumulation frame count of 2, producing a total of 1440 scan images. To obtain detailed information on the pore distribution within the sandstone samples, the grayscale images were binarized and combined with three-dimensional digital-imaging technology to generate a digital model containing only the pores and the solid framework. Subsequently, the pores were represented by small cubic units, each with a side length of one pixel centered on each pixel point. The maximum sphere algorithm was employed to extract pores, providing detailed information on the pore size, pore size distribution, and pore coordination number for the three-dimensional model.

## 3. Results and Discussion

### 3.1. Residual Oil Distribution Post-Polymer Flooding

According to the research findings of Li et al. [42], influenced by the interfacial tension, the displacement intensity, and the microstructure of the reservoir, the remaining oil in the reservoir can be classified into three primary categories: bound state, semi-bound state, and free state.

**Figure 5:**
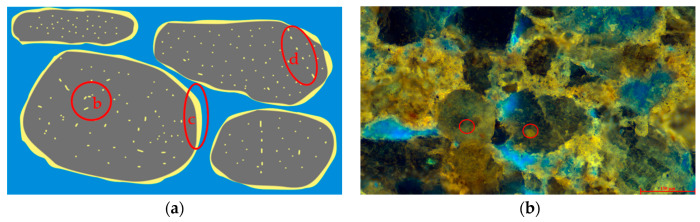

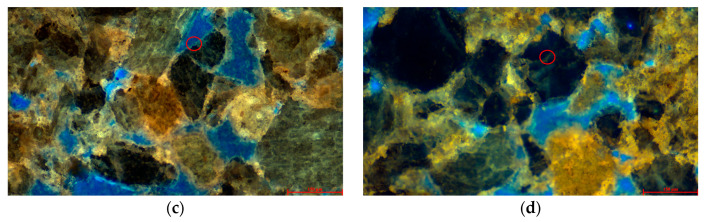
Morphology of remaining oil in a bound state. (**a**) Bound residual oil occurrence pattern diagram. (**b**) Particle adsorbent residual oil. (**c**) Pore surface film remaining oil. (**d**) Slit residual oil (black represents rock, blue represents water, and yellow represents crude oil).

Bound-state residual oil, affected by interfacial tension, is further divided into three subtypes: pore-surface film, particle-adsorbed, and slit-confined, as illustrated in Figure 5. Pore-surface film residual oil forms a thin layer of oil around the pore walls, as shown in Figure 5c. This occurs when the oil phase comes into contact with the surface of particles within the pores. Due to the imbalance in the molecular structure within the particles and the uneven molecular force field at the surface layer, significant natural interfacial energy arises between the oil phase and the particles, resulting in the adsorption of the oil and gas.

Particle-adsorbed residual oil appears as spot-like or regionally continuous accumulations on the surfaces of particles, as shown in Figure 5b. This is particularly evident in clay mineral particles. Some mineral particles exhibit weak dissolution, creating non-conductive pores where oil can enter but not exit.

Slit-confined residual oil is trapped within the microfractures between particles or within the particles themselves, as depicted in Figure 5d. The resistance due to interfacial tension and capillary forces makes it difficult for this oil phase to be displaced.

**Figure 6:**
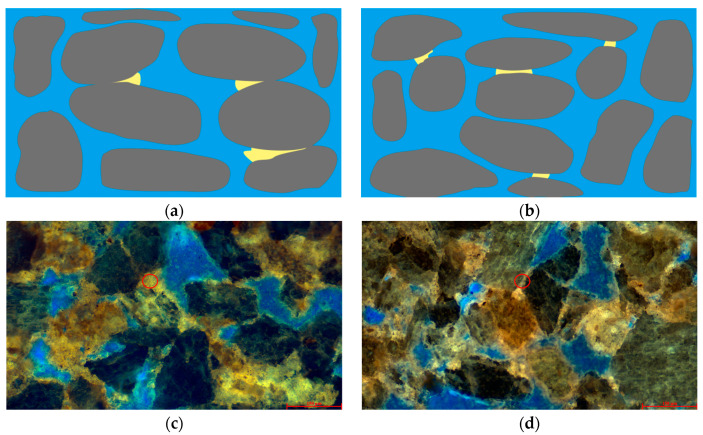
Semi-bound remaining oil morphology diagram. (**a**) Corner pattern of remaining oil occurrence. (**b**) Throat pattern of residual oil occurrence. (**c**) Corner residual oil. (**d**) Laryngeal residual oil (black represents rock, blue represents water, and yellow represents crude oil).

Residual oil in the semi-bound state is classified into two forms: corner-bound and throat-bound, as illustrated in Figure 6. Corner-bound residual oil primarily resides at the corners of pores (Figure 6c). This formation occurs due to two main reasons: firstly, the surface tension and capillary action of rock particles anchor the oil phase in the pore corners; and secondly, insufficient displacement intensity results in incomplete fluid diffusion within the pores, making it difficult to displace the oil trapped in these corners.

Throat-bound residual oil remains within the pore throats (Figure 6d). Due to capillary resistance, this type of residual oil typically accumulates in smaller throats. During the displacement process, the aqueous-phase fluid preferentially flows through larger pores and throats, forming dominant pathways, which render the oil in the smaller throats challenging to effectively displace.

**Figure 7:**
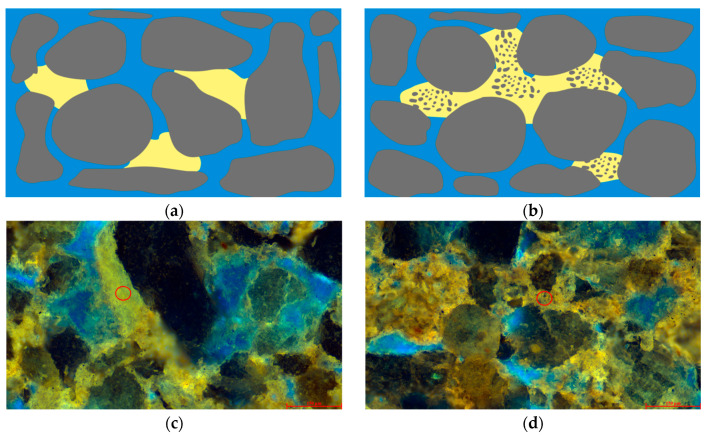
Free remaining oil morphology diagram. (**a**) Cluster residual oil occurrence pattern. (**b**) Intergranular adsorption residual oil occurrence pattern diagram. (**c**) Clusters of residual oil. (**d**) Intergranular adsorption of residual oil (black represents rock, blue represents water, and yellow represents crude oil).

Free-state residual oil can be categorized into two forms: clustered and interparticle adsorption, as illustrated in Figure 7. Clustered residual oil primarily exhibits a lamellar structure within the pores (Figure 7c). Influenced by the displacement intensity and capillary action, portions of the oil phase within the pores, especially in those containing fine throats, are not effectively displaced.

Interparticle-adsorbed residual oil resides in the interstices between fine particles within the pores (Figure 7d). The interfacial tension among these numerous micro-particles is relatively high, granting them a stronger adsorption capacity for oil and gas. Additionally, the small pore throat sizes formed by these fine particles make it challenging for conventional displacement methods to effectively mobilize these oil phases.

### 3.2. Quantitative Analysis of Microscopic Residual Oil

**Figure 8:**
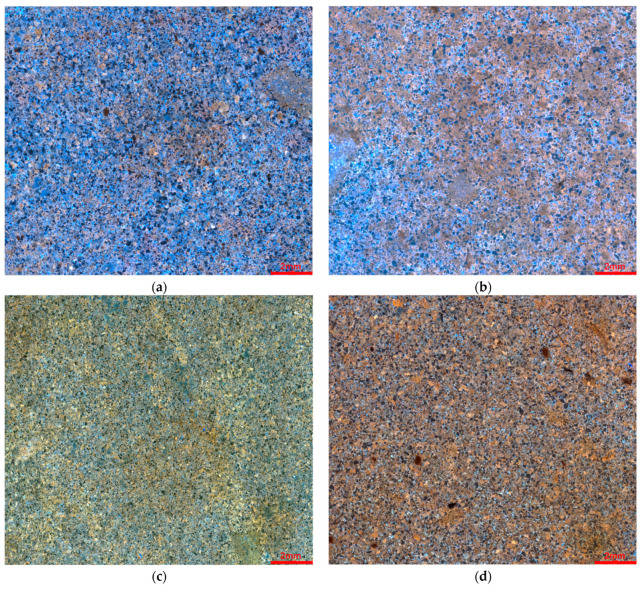

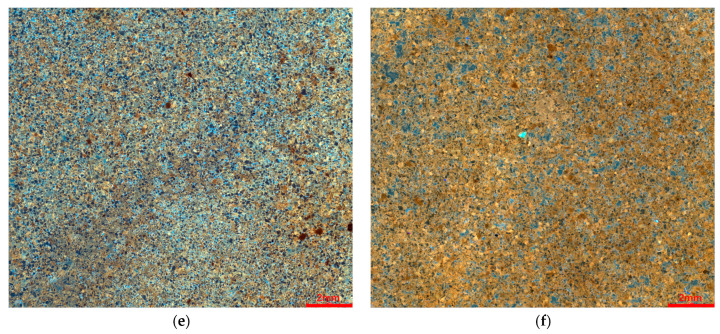
Distribution of oil–water characteristics of reservoirs with different permeability. (**a**) Oil and water distribution in the whole view of sample 1. (**b**) Oil and water distribution in the whole view of sample 2. (**c**) Oil and water distribution in the whole view of sample 3. (**d**) Oil and water distribution in the whole view of sample 4. (**e**) Oil and water distribution in the whole view of sample 5. (**f**) Oil and water distribution in the whole view of sample 6 (black represents rock, blue represents water, and yellow represents crude oil).

By stitching together 256 images (with a resolution of 3840 × 2160), a large field-of-view image was created, as shown in Figure 8. Under varying degrees of water washing, the effect of polymer flooding on rock samples subjected to intense washing was markedly superior to those not subjected to such treatment. Among the high-permeability rock samples, sample 1, which underwent intensive water washing, exhibited a significant reduction in the residual oil content, achieving a water saturation of 52.1%, with a relatively uniform distribution of oil and water, as illustrated in Figure 8a. In contrast, the thin section of sample 2, which did not undergo intensive washing, displayed an uneven distribution of the residual oil, appearing as distinct patches of blue and brown, with an irregular oil-water distribution.

As the permeability of the rock decreases, the distribution of the residual oil becomes more pronounced, and the influence of water correspondingly diminishes, as depicted in Figure 8b. Sample 3 displayed a water saturation of 42.9%, with thin sections showing a bright yellow color, primarily composed of bituminous oil, as shown in Figure 8c. Sample 4 exhibited a further reduced water saturation of 30.9%, with the thin sections appearing brown, predominantly composed of colloidal and bituminous asphalt, as depicted in Figure 8d.

In the low-permeability rock samples, the extent of the water invasion was minimal. Sample 5 had a water saturation of 34.1%, displaying a light yellow hue with some orange–yellow regions, mainly containing bituminous oil with some colloidal asphalt, as illustrated in Figure 8e. Sample 6 had the lowest water saturation at 22.6%, presenting a reddish–brown color, primarily composed of bituminous asphalt, as shown in Figure 8f.

Through the application of fluorescence analysis technology on the rock thin sections, a quantitative analysis of the various residual oil occurrence forms was conducted, as shown in Table 3. Following the polymer-flooding process, in the high-permeability rock samples, more than 50% of the residual oil is in the bound state, primarily manifesting as particle-adsorbed residual oil. In the medium-permeability rock samples, over 49% of the residual oil is in the free state, predominantly in the form of interparticle-adsorbed residual oil. Conversely, in the low-permeability rock samples, more than 47% of the residual oil is in the bound state, mainly as particle-adsorbed residual oil. Future polymer-flooding strategies should prioritize the optimization of particle-adsorbed residual oil in high- and low-permeability reservoirs, while in medium-permeability reservoirs, the focus should be on the development and optimization of interparticle-adsorbed residual oil. The primary target for polymer development should thus be medium- to low-permeability reservoirs.

### 3.3. Distribution Characteristics of Residual Oil Components in Microscopic Pores

**Figure 9:**
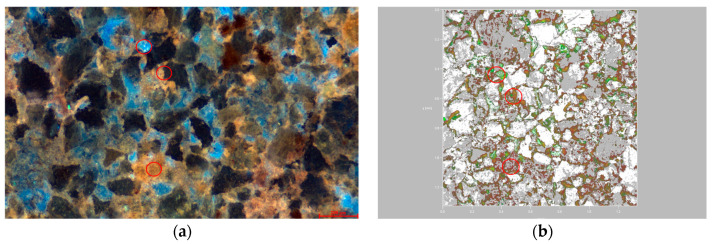
Occurrence state of microscopic residual oil in the high-permeability reservoir after polymer flooding. (**a**) Remaining oil occurrence state of the high-permeability reservoir. (**b**) Occurrence state of light and heavy components of remaining oil in the high-permeability reservoir.

Using laser scanning confocal microscopy, we performed layer-by-layer scanning on samples with varying permeability, ultimately obtaining three-dimensional visual images. These images allow for an intuitive analysis of the distribution patterns of the light and heavy components of residual oil within the pore structure, as well as the influence of the pore structure on the distribution of residual oil. As shown in Figure 9b, white represents transparent mineral particles, green indicates light components, and red denotes heavy components. According to the distribution of residual oil in the high-permeability reservoirs after polymer flooding, as depicted in Figure 9, the segregation of light and heavy components in the oil is quite distinct, with a light-to-heavy component ratio of 0.89. It was observed that in water-bearing pores, the main components in contact with the rock samples were predominantly light (green), and these were primarily isolated. Most water-bearing pores in the high-permeability reservoirs contained a significant amount of light components, mainly aromatics, with no heavy components detected. This suggests that these light components migrated here post-polymer flooding. Conversely, heavy components (red) were primarily found on the surface of mineral particles and in non-water-bearing pores, distributed widely and continuously. This extensive distribution is attributed to the hydrophobic surface activity of heavy components, mainly comprising colloidal and bituminous asphalt, which tend to align directionally on the surfaces of mineral particles.

**Figure 10:**
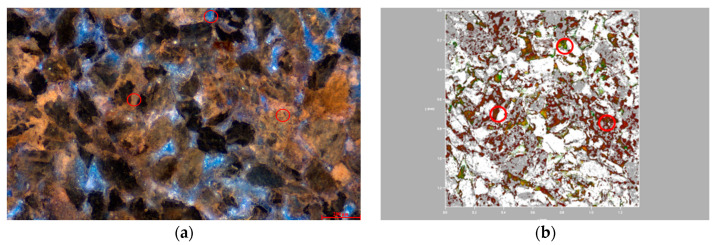
Occurrence state of micro residual oil in the medium-permeability reservoir after polymer flooding. (**a**) State of remaining oil occurrence in the medium-permeability reservoir. (**b**) Occurrence state of light and heavy components of remaining oil in the medium-permeability reservoir.

In the medium-permeability reservoirs post-polymer flooding, as illustrated in Figure 10, heavy components are predominantly distributed in contiguous patches, while light components are distinctly isolated in localized concentrations. The light-to-heavy component ratio is 0.93. Within water-bearing pores, light components dominate, coexisting with heavy components in a clustered formation. This coexistence is likely due to the insufficient displacement force. In the pores between larger particles, residual oil is mainly composed of heavy components, with light components also present in clustered formations. Conversely, oil and gas adsorbed on the surfaces of fine particles and within the interstices are primarily heavy components, shown in red. The small pores formed by these fine particles experience significant capillary forces and have a large contact area between the particles and the oil phase, causing heavy components to remain trapped through weak interactions like van der Waals forces.

**Figure 11:**
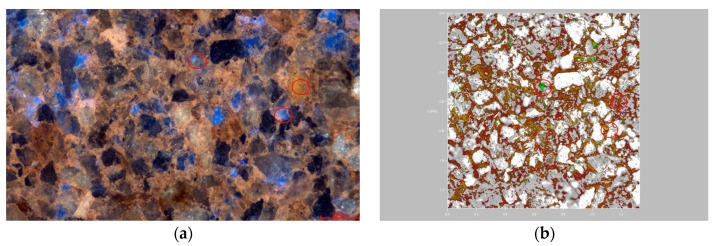
Microscopic residual oil occurrence in the low-permeability reservoir after polymer flooding. (**a**) State of remaining oil occurrence in the low-permeability reservoir. (**b**) Occurrence state of light and heavy components of remaining oil in the low-permeability reservoir.

In the low-permeability reservoirs post-polymer flooding, as depicted in Figure 11, the smaller particle size compared to the medium- and high-permeability reservoirs results in the formation of smaller pores. The capillary forces within these small pores make oil-phase displacement difficult. Consequently, the light components of the residual oil are distinctly isolated, while the heavy components are continuously scattered in patch-like formations. The light-to-heavy component ratio is 0.84. In most water-bearing pores, the oil phase’s light and heavy components coexist in a balanced manner, while a few water-bearing pores are dominated by light components. Clustered residual oil exists with a balanced coexistence of light and heavy components, whereas particle-adsorbed residual oil predominantly consists of heavy components with minor amounts of light components.

### 3.4. The Influence of Clay Particles and Clay Minerals on the Distribution of Microscopic Residual Oil Post-Polymer Flooding

The content of clay minerals plays a critical role in controlling the distribution of residual oil and significantly impacts the efficiency of polymer flooding. Different characteristics of clay minerals affect reservoir properties in various ways. Erosion-generated detrital particles and clay minerals often mix to form clay particles, which, due to their fineness, fill the spaces between sand grains, thereby reducing the effective pore space. The adsorptive nature of clay particles impedes oil-phase displacement, leading to the formation of interparticle-adsorbed residual oil. Additionally, the presence of clay particles can reduce the throat radii or cause pore closure due to their swelling properties, further hindering oil migration.

Therefore, when designing polymer-flooding schemes, it is essential to fully consider the influence of clay particles and clay minerals on the efficiency of polymer displacement. As shown in Table 4, the reservoir primarily contains kaolinite, with a content of 48% or higher, followed by mixed-layer illite-smectite, with a content ranging from 28% to 37%. The contents of illite and chlorite are relatively lower.

**Figure 12:**
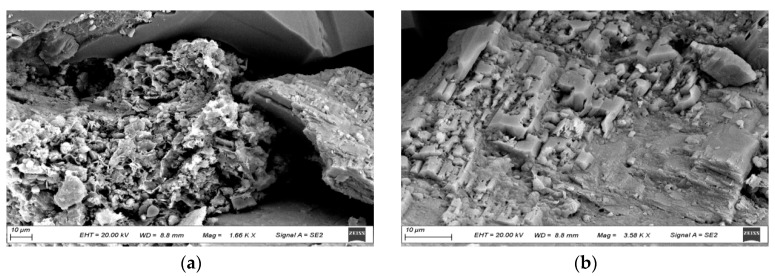

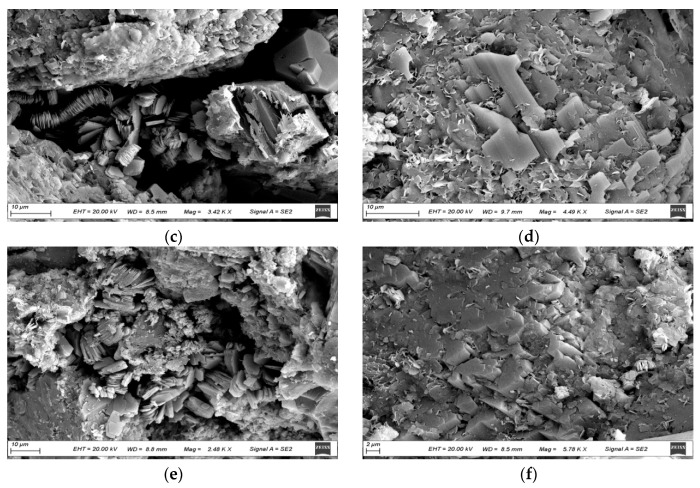
Mirror images of reservoirs with different permeability after polymer flooding. (**a**) Intergranular pores in high-permeability reservoirs. (**b**) Clay minerals lining grain surfaces in high-permeability reservoirs. (**c**) Intergranular pores in medium-permeability reservoirs. (**d**) Clay minerals lining grain surfaces in medium-permeability reservoirs. (**e**) Intergranular pores in low-permeability reservoirs. (**f**) Clay minerals lining grain surfaces in low-permeability reservoirs.

Simultaneously, as observed through backscattered scanning electron microscopy in Figure 12, kaolinite in untreated reservoirs typically appears in book-like or vermicular forms. In high-permeability reservoirs, the clay content is minimal, and the kaolinite within the pore throats appears disordered, indicating a more effective displacement process. The presence of clay minerals such as illite-smectite on the surface of feldspar particles is a primary factor contributing to particle-adsorbed residual oil.

In medium-permeability reservoirs, there is an increase in clay particles, with some pore throats filled with book-like kaolinite, indicating weaker displacement effects. The surfaces of particles in these reservoirs are often lined with chlorite and illite-smectite clay minerals. Chlorite, with its interlayer distribution, offers a large specific surface area and high surface activity, thus exerting a stronger adsorptive effect on the oil phase.

In low-permeability reservoirs, the clay content is the highest, with most pore throats filled with book-like kaolinite. The narrow pore throats, filled with clay minerals, hinder effective displacement. The surfaces of particles in these reservoirs are often lined with kaolinite and chlorite, further complicating oil migration due to their high surface activity and substantial adsorption capacity.

### 3.5. The Impact of the Pore–Throat Structure on the Distribution of Microscopic Residual Oil

**Figure 13:**
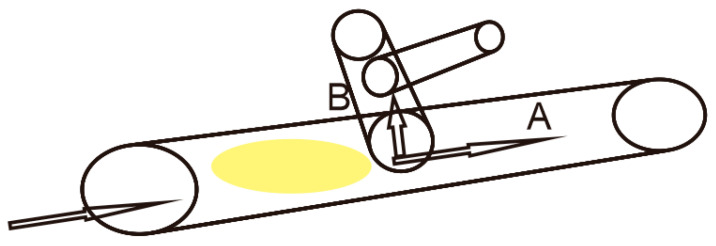
Distribution of residual oil in capillaries.

Given the complexity of the internal spatial structure of the reservoir, studying the micro-pore structure is crucial for understanding the efficiency of residual oil displacement. During the migration of the oil phase, it is influenced by the driving force, gravity, friction, capillary force, and viscosity. In porous media reservoirs, when the oil-phase radius is smaller than the pore radius, as illustrated by pore A in Figure 13 the effects of friction and the Jamin effect are minimal, resulting in lower migration resistance. Conversely, when the oil-phase radius exceeds the pore radius, the friction and Jamin effect significantly increase the resistance, as shown between pore A and pore B in Figure 13. When the displacement force is insufficient, the oil phase struggles to migrate to pore B.

**Figure 14:**
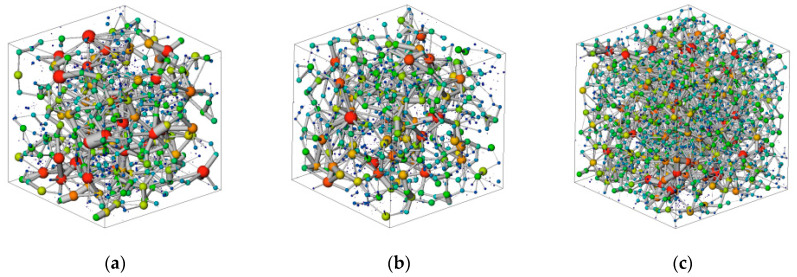
Pore network model of reservoirs with different permeability. (**a**) Pore network model of high-permeability reservoirs. (**b**) Pore network model of medium-permeability reservoirs. (**c**) Pore network model of low-permeability reservoirs.

Micro-CT scanning was performed on samples from reservoirs with different permeabilities to extract the pore and throat spatial network structure. In the digitized three-dimensional core analysis, a set of maximally inscribed spheres of varying sizes was constructed to depict the structure of the pore space. These spheres, though differing in size and partially overlapping, collectively form a complex group of spherical structures. By identifying the local maximum spheres and the smallest sphere between two adjacent maximum spheres, the critical components of the pore network, namely “pores” and “throats”, are clearly defined. This process transforms the intricate three-dimensional core pore space into a simplified model known as the “pore-throat model”, which is composed of basic elements of “pores” and “throats.” As shown in Figure 14a,b, the pore–throat structure in medium- to high-permeability reservoirs is relatively simple, whereas in low-permeability reservoirs, the pore–throat structure is more complex, as depicted in Figure 14c.

The pore network model primarily examines two elements: pores and throats, focusing on parameters such as the pore radius (PR), pore volume (PV), coordination number (PC), and pore shape factor (PS). For throats, the key parameters include the throat radius (TR), throat volume (TV), throat length (TL), and throat shape factor (TS). The relationship function between two random variables, S and T, serves as a measure of the linear relationship between these variables. For the sequence{(*s*_1_,*t*_1_),(*s*_2_,*t*_2_), …, (*s_n_*,*t_n_*)}, the correlation function can be defined as follows:

(1) $$r_{\left(S,T\right)}=\frac{n\sum_{i=1}^{n}s_{i}t_{i}-\sum_{i=1}^{n}s_{i}\sum_{i=1}^{n}t_{i}}{\sqrt{n\sum_{i=1}^{n}s_{i}{\;\;}^{2}-{\left(\sum_{i=1}^{n}s_{i}\right)}^{2}}\sqrt{n\sum_{i=1}^{n}t_{i}{\;\;}^{2}-{\left(\sum_{i=1}^{n}t_{i}\right)}^{2}}}$$

The coordination number in the pore network model is a crucial parameter, reflecting the degree of connectivity within the pore space, which significantly impacts the permeability. Analyzing the distribution of the coordination number effectively evaluates the topological properties of the rock. Therefore, particular attention must be paid to the coordination number when constructing random pore network models. The radii of the pore-throat elements in the original pore network model are evenly divided into m parts, such that *r_min_* = *r*_1_ < … *r_m_* = *r_max_*. The corresponding connectivity functions *X_v_*(*r*_1_), …, *X_v_*(*r_m_*) are then calculated. The global connectivity of the pores can be represented by the connectivity function as follows:

(2) $$\chi_{v}(r)=\frac{N_{p}(r)-N_{t}(r)}{V}$$

In this context, *N_p_*(*r*) represents the number of pore spheres in the original pore network model with a radius greater than *r*, while *N_t_*(*r*) denotes the number of throats with a radius greater than *r*. *V* indicates the volume of the original pore network model in cubic meters.

Calculating the structural parameters of the digital core reveals that the pore–throat structures vary across reservoirs with different permeabilities, as shown in Table 5. High-permeability reservoirs exhibit the largest pore and throat radii, with the highest proportion of pores exceeding 10 μm in radius and a uniform distribution, making them the primary migration channels. These reservoirs have the fewest isolated pores and the highest proportion of pores connected to more than three throats, resulting in an optimal pore–throat structure and connectivity. Consequently, high-permeability reservoirs demonstrate the most effective mobilization of residual oil during displacement, with the overall displacement pattern resembling a network, leaving relatively more bound residual oil.

Medium-permeability reservoirs have slightly inferior pore–-throat structures, with 41.5% of the pores having a radius greater than 10 μm. These reservoirs predominantly feature medium to large pores. As the pore radius decreases, their storage capacity diminishes. The coordination number ratio indicates further reduced pore–throat connectivity. Due to the displacement dynamics, some pores remain unaffected, leading to finger-like flow patterns and higher residual free oil content in localized areas.

Low-permeability reservoirs have the poorest pore structure, with only 28.3% of the pores exceeding a 10 μm radius and an average pore radius of 15.3 μm. This variability results in a heterogeneous spatial structure. In the presence of a few large pores, fluid tends to flow through these dominant channels during displacement, resulting in less effective displacement and a finger-like displacement pattern. Additionally, weaker dissolution in these reservoirs leads to numerous isolated pores formed by incomplete feldspar dissolution. The lack of connectivity hinders the mobilization of residual oil in these pores, and the dominant flow paths cause most pores to remain unaffected, resulting in the most pronounced residual oil retention.

## 4. Conclusions

This study focuses on the distribution patterns and occurrence states of residual oil in reservoirs with varying permeabilities following polymer flooding. It also analyzes the factors influencing the migration of residual oil impacted by polymer flooding, providing scientific and reliable data for adjusting polymer-flooding strategies in future extraction processes. The primary conclusions are as follows:

(1) Post-polymer flooding, residual oil at the microscopic level predominantly exists in seven distinct forms: bound state (particle-adsorbed, pore surface film, and slit-confined), semi-bound state (corner-bound and throat-bound), and free state (clustered and intergranular adsorbed). Compared to reservoirs that have not undergone intensive water washing, those subjected to intensive water washing show a 5.8% reduction in free-state residual oil. In the study area, free-state residual oil primarily exists as intergranular-adsorbed oil, which is a target for further exploitation. This can be achieved by adding surfactants to the polymer solution, the reducing oil–water interfacial tension, and minimizing the adsorption of oil on particle surfaces, thereby facilitating the release of oil trapped between fine particles.
(2) In high-permeability reservoirs, residual oil primarily exists in a state of particle adsorption. The localized enrichment of residual oil in reservoirs not subjected to enhanced water washing is more pronounced, making these reservoirs potential targets for development. In medium-permeability reservoirs, there is a greater presence of free-state residual oil, and the oil content of the reservoirs is higher, thus becoming the primary focus for adjusting post-polymer-flooding production relations. Although low-permeability reservoirs are rich in oil, their heterogeneous spatial structure increases the difficulty of development. For clustered and corner residual oil, the injection scheme should be optimized, including the injection rate, concentration, and volume, to ensure uniform polymer distribution, maximize sweep volume, and enhance oil displacement efficiency.
(3) When considering post-polymer-flooding measures, attention should be paid to changes in the crude oil composition and distribution characteristics. Selecting the most suitable polymer type and molecular weight is crucial to ensure optimal solubility and thickening effects. In high-permeability reservoirs, due to a higher degree of water flooding, light components are relatively scarce. In medium-permeability reservoirs, the displacement degree is lower, with light components dominating the water-bearing pores, while the majority of other pores contain heavy components. In low-permeability reservoirs, a few water-bearing pores are dominated by light components, most water-bearing pores exhibit a balance of light and heavy components, and overall, the reservoir prominently features heavy components.
(4) The content and distribution of clay minerals significantly influence the occurrence forms of microscopic residual oil and should be a key consideration in the later adjustment of polymer-flooding schemes. Kaolinite accounts for about 50% of the clay mineral content in the reservoir. The presence of these clay minerals and argillaceous particles can block pores and exhibit strong adsorption effects on the reservoir, thereby impacting oil migration.
(5) The microscopic pore structure is also a critical factor affecting the distribution and morphology of residual oil post-polymer flooding. The migration of oil in the reservoir is influenced by factors such as the displacement force, pore–throat radius, pore–throat connectivity, friction, and the Jamin effect. In the later adjustment of polymer-flooding schemes, enhancing the displacement force and adding appropriate amounts of surfactants to the polymer solution can reduce the interfacial tension, thereby improving the recovery rate of the reservoir.

## Figures and Tables

**Table 1:** Basic parameters of the experimental cores.

Core Number	Porosity (%)	Permeability (mD)	Strong Washing Degree (%)	Remaining Oil Saturation (%)
Rock Core 1	30.63	1172.0	1.9	11
Rock Core 2	33.48	1137.0	0.0	22
Rock Core 3	30.87	469.9	1.9	17
Rock Core 4	29.63	488.1	0.0	22
Rock Core 5	28.81	177.0	1.9	16
Rock Core 6	28.57	146.8	0.0	14

**Table 2:** Color of the crude oil components under ultraviolet light.

Crude Oil Component	Luminous Color
Aromatics	Blue, blue–white, Light blue–white
Oily asphalt	Yellow, brown, light yellow, yellow–white, yellow–green, green, light yellow–green, yellow–green, light green, blue–green, light blue–green
Gum asphalt	Orange, orange, orange–brown, light orange, light orange–brown, light orange–yellow
Asphaltic pitch	Red, brown, light orange–brown, light brown, orange–brown, yellow–brown, light yellow–brown
Carbonaceous bitumen	Non-luminescence

**Table 3:** Occurrence states of residual oil in reservoirs with different permeabilities post-polymer flooding.

Core Number	Bound (%)			Semi-Bound (%)		Free (%)		Water Saturation (%)
	Pore Surface Membrane	Granular Adsorption	Slit	Corner	Throat	Cluster	Intergranular Adsorption	
Rock Core 1	13.60	38.53	0.31	2.19	1.94	6.10	37.27	52.10
Rock Core 2	10.97	39.17	0.41	2.15	1.65	8.24	37.41	43.80
Rock Core 3	2.17	46.25	0.62	0.91	0.44	3.06	46.56	42.90
Rock Core 4	3.76	29.66	0.77	5.01	2.55	23.27	34.99	30.90
Rock Core 5	13.17	32.89	2.98	4.93	4.17	21.28	20.59	34.10
Rock Core 6	10.33	36.32	0.71	2.52	1.72	9.93	38.47	22.60

**Table 4:** Average content table of mud and clay minerals in different-permeability reservoirs.

Reservoir Type	Muddy Content (%)	Clay Content (%)	Relative Clay Mineral Content (%)			
			Aemon Mixed Layer	Illite	Kaolinite	Chlorite
High-permeability reservoir	3.25	5.2	34	11	48	7
Medium-permeability reservoir	10.07	4.3	28	13	51	8
Low-permeability reservoir	12.80	4.7	37	9	48	6

**Table 5:** Pore–throat structure parameters in reservoirs with different permeabilities.

Reservoir Type	Average Pore Radius (µm)	Mean Throat Radius (µm)	Pore Radius Distribution Frequency (%)		Coordination Number Ratio (%)		
			Radius > 10 µm	Radius < 10 µm	0	1–3	>3
High-permeability reservoir	26.1	10.8	51.2	48.8	17.58	60.59	21.84
Medium-permeability reservoir	20.9	8.9	41.5	58.5	19.11	63.87	17.02
Low-permeability reservoir	15.3	6.5	28.3	72.7	28.39	64.29	7.32

## Data Availability

Data are contained within the article.
